# Transcriptional Modulation in Grapevine by a Biostimulant Treatment for Improved Plant Resilience to Stress Events

**DOI:** 10.3390/plants15020283

**Published:** 2026-01-17

**Authors:** Asia Mostacci, Domenico Di Cosmo, Ornella Incerti, Antonio Ippolito, Rita Milvia De Miccolis Angelini, Simona Marianna Sanzani

**Affiliations:** Department of Soil, Plant and Food Sciences, University of Bari Aldo Moro, via Amendola 165/A, 70126 Bari, Italy; a.mostacci@phd.uniba.it (A.M.); domenico.dicosmo@uniba.it (D.D.C.); ornella.incerti@uniba.it (O.I.); antonio.ippolito@uniba.it (A.I.); ritamilvia.demiccolisangelini@uniba.it (R.M.D.M.A.)

**Keywords:** plant hydrolysates, *Vitis vinifera* L., RNA-seq, MapMan, time-course study, biostimulants, stress condition

## Abstract

Grapevine (*Vitis vinifera* L.) is a globally significant crop increasingly affected by a variety of biotic and abiotic stresses. Plant biostimulants offer a promising approach to enhance plant resilience by modulating key physiological and metabolic processes. This study aimed to demonstrate that the preventive application of a *Fabaceae*-based biostimulant can prime grapevine defense pathways, thereby improving plants’ ability to endure potential stress conditions. Indeed, resistance to both biotic and abiotic stresses in plants involves common pathways, including Ca^2+^ and ROS signaling, MAPK cascades, hormone cross-talk, transcription factor activation, and induction of defense genes. Grapevine leaves were subjected to high-throughput transcriptomic analysis coupled with qPCR validation 6 and 24 h following treatment application. Differentially expressed genes were visualized using MapMan to identify the major metabolic and signaling pathways responsive to the treatment. This integrative analysis revealed several defense-related pathways triggered by the biostimulant, with representative protein families showing both up- and downregulation across key functional categories. Overall, the results indicate that a wider array of pathways associated with stress tolerance and growth regulation were stimulated in treated plants compared to untreated controls. These findings support the conclusion that a preventive biostimulant application can effectively prime grapevine metabolism, enhancing its preparation to cope with forthcoming environmental challenges.

## 1. Introduction

Grapevine (*Vitis vinifera* L.) is a major global fruit crop cultivated for fresh consumption and processed in a variety of products, such as wine, raisin, juice, and vinegar. In 2024, with an average yield of 10.9 t per ha, the world’s vineyards, equaling around 7.1 mha, produced a total of 77.7 mt of fresh grapes [1,2]. The top cultivating areas are in Europe, namely Spain (930 kha), France (783 kha), and Italy (728 kha); in Asia, namely China (753 kha); and in America, namely Chile (166 kha) [2]. Grape cultivation is severely affected by changing climatic conditions and phytopathogens, such as *Botrytis cinerea*, *Plasmopara viticola*, and *Erysiphe necator*, which cause gray mold, downy mildew, and powdery mildew, respectively, leading to significant yield and quality losses and necessitating intensive chemical control [3,4,5,6]. Fungicides are widely used against grape diseases, but concerns over their environmental and health impacts have driven interest in developing eco-friendly alternatives to conventional plant protection products [7]. Natural compounds including peptides and protein hydrolysates showed strong potential both as biostimulants [8,9] and enhancers of grapevine defense responses against stresses, including pathogen attacks [10]. The category of resistance inducers includes natural elicitors [11], polyols [12], plant extracts [13], Plant Growth-Promoting Rhizobacteria (PGPR), and humic acids [14]. In some previous investigations [15,16,17], a soybean protein hydrolysate, produced for pharmaceutical use, proved to effectively induce defense reactions against water stress, gray mold, and downy mildew in grapevine tissue, including signal transduction, priming of mitogen-activated protein kinases (MAPKs), differential expression of a selection of pathogenesis-related (PR) proteins, and the synthesis of phenylpropanoid-derived compounds as stilbenes, which represent the major antimicrobial compounds in grapevine [18]. This hydrolysate powder was later formulated and further tested as a biostimulant on lettuce plants, revealing the ability to increase plants’ resistance to stresses [19]. However, a better and more comprehensive insight into the genetic responses to elicitation by the formulate was still needed to set up more effective application strategies. In the present investigation, a transcriptomic analysis of the metabolic pathways activated by the above-mentioned formulate was conducted on the leaves of grapevine plantlets. The grapevine was selected considering the previous data [15,16,17] and the availability of well-annotated grapevine genomes.

The rapid development of next-generation sequencing technologies and consequent availability of grapevine genomes have greatly enhanced gene discovery and functional studies. For instance, Vannozzi et al. [20] provided a complete characterization of the stilbene synthases multigenic family in grapevine, integrated with a comprehensive set of gene expression analyses, including both healthy tissues at different developmental stages and tissues exposed to biotic (downy mildew) or abiotic (wounding and UV-C exposure) stresses. Similarly, Sun et al. [21] investigated the regulations of grapevine stress-associated proteins in the presence of stress based on transcriptomic analysis and discussed them in terms of signaling cross-talking at cellular and organic levels. In fact, since plant response to abiotic stress must be coordinated with growth and development, the consequent host physiological status might confer susceptibility/tolerance through biotic/abiotic signaling cross-talking under biotic stress. Moreover, the application of omics technologies provided a better understanding of plant–pathogen interactions in crop plants, like grapevine, which is necessary to highlight how pathogens infect the plant and how host defenses are activated and might be enhanced [22]. This, in turn, might help in finding biotechnological solutions to reduce the economic losses associated with diseases, thus reducing the need for conventional chemical agents.

Several studies have sought to investigate differences in gene expressions between *Vitis* species exposed to a variety of biotic and abiotic stresses using DNA microarrays. However, this technique presents many limitations in analyzing the total transcriptome, especially with the highly complex *V. vinifera* genome [4,23,24]. Thus, in the present investigation, we used high-throughput deep-sequencing technology to characterize the global transcriptional dynamics associated with the application of the soybean protein hydrolysate formulation to grapevine. The global transcriptional profile of grapevine leaves treated with the formulation was analyzed to identify the major groups of genes modulated as compared to untreated samples. The most highly represented and meaningful functional categories were described and discussed.

## 2. Materials and Methods

### 2.1. Plant Elicitor Preparation

A commercial formulate based on a *Fabaceae* protein hydrolysate (AgricostanD, AgriD) was provided by A. Costantino SpA (Favria, Tourin, Italy). The working solution was prepared 24 h before use, at a 2.5 mL/L concentration, and stored at 4 °C until use.

### 2.2. Experimental Set Up

For RNA-Seq assays, 18 grapevine plantlets (8 weeks old) of *V. vinifera* L. cv. Corvina (own-rooted) in 1.2 L pots were located in a greenhouse at 24/18 °C (day/night) with a photoperiod of 16 h, a light intensity of 600 µmol/m^2^·s, and a relative humidity (RH) of 70 ± 10%. Plants were randomly divided into two blocks, composed of three replicates made of three plants each. After 48 h and a single watering, the first block was sprayed until dripping with the formulation, and the second was sprayed with tap water. The experimental design was completely randomized for both plant sets. Leaves were collected 6 and 24 h post-treatment (HPT), immediately frozen in liquid nitrogen, grounded, and kept at −80 °C until RNA extraction. These harvesting times were selected based on previous differential expression assays [15]. For each time point and treatment, leaves from one plant per biological replicates (3 plants in total) were sampled and pooled prior to RNA extraction. Leaves were collected from the whole plant.

### 2.3. Extraction of Total RNA, cDNA Library Preparation and Illumina Sequencing

Total RNA was isolated from 150 mg of plant tissue and subsequently treated with DNase using the Plant DNA/RNA Isolation Kit (Norgen, Thorold, ON, Canada) as suggested by the manufacturer. The integrity of the RNA was assessed using a Bioanalyser 2100 (Agilent Technologies, Santa Clara, CA, USA). Additionally, RNA concentration and purity were measured with both a NanoDrop 2000 spectrophotometer (Thermo Scientific, Waltham, MA, USA) and a Qubit 2.0 fluorometer (Life Technologies, Carlsbad, CA, USA). For the construction of complementary DNA (cDNA) libraries and RNA sequencing, four poly(A)^+^ mRNA-seq libraries (see Table 1) were prepared using the TruSeq™ RNA Sample Preparation Kit (Illumina Inc., San Diego, CA, USA), starting from 4 μg of total RNA following the low-throughput protocol recommended by the manufacturer. The synthesized cDNAs were size verified by electrophoresis on a 1% agarose gel, confirming an approximate fragment size of 240 base pairs. Library quantification was performed using both the NanoDrop 2000 (Thermo Scientific) and Qubit 2.0 instruments (Life Technologies). The libraries were then normalized to a concentration of 10 pM, as advised by the manufacturer, pooled in an eight-plex format, and loaded onto the flow cell. Cluster generation and hybridization were carried out on a cBot System employing the TruSeq SR Cluster Kit v3 (Illumina Inc.). Sequencing was conducted on an Illumina HiScanSQ platform using the TruSeq SBS Kit v3 (Illumina Inc.), employing a single-read 50 base pair (1 × 50 bp) sequencing strategy.

### 2.4. Computational Analysis and Functional Assignments of Differentially Expressed Genes

Base calling was performed using the Illumina CASAVA software (version 1.8.1.) and checked for quality using FastX-tools (http://hannonlab.cshl.edu/fastx_toolkit/, accessed 8 September 2025). Raw RNA-Seq raw were deposited in figshare (https://figshare.com/s/8451b9e2b78bd2bcece2, accessed 22 Dicember 2025). Trimmed reads were mapped against the most recent *V. vinifera* reference genome (cv. Pinot Noir 40024; GenBank assembly GCA_030704535.1) retrieved from the National Centre for Biotechnology Information (https://www.ncbi.nlm.nih.gov/datasets/genome/GCF_030704535.1, accessed 22 September 2025), and differential gene expression analysis, incorporating False Discovery Rate (FDR) correction, was performed using CLC Genomic Workbench v. 25.0.1 (QIAGEN, Hilden, Germany). It was assumed that genes with comparable average expression levels exhibited similar variability. Absolute values of fold change > 2 and FDR-adjusted *p*-values ≤ 0.05 were used as thresholds to judge the significance of gene expression difference.

Functional assignment was performed using Omicsbox v 3.4.5 (BioBam, Valencia, Spain) on the dataset to predict categories of transcriptional modulation induced by formulate treatment.

For data analysis, MapMan software version 3.6.0RC1 (http://mapman.gabipd.org/web/guest/mapman, accessed 6 October 2025), which displays metabolic maps, highlighting important pathways and giving an overview of induced processes [25,26], was used.

The Phytozome v9.0 *V. vinifera* genome (Vvinifera_145_ v1.fa.gz) was used as mapping reference. Expression data (fold change) were analyzed to visualize the metabolic pathways up/downregulated by formulate application at 6 and 24 HPT.

### 2.5. Validation of RNA-Seq Data by qPCR

To validate the expression profiles obtained by RNA-Seq, qPCR was performed on six genes randomly selected for high or low expression levels. The sequences of the primer pairs used are reported in Table 2. For each primer pair, calibration curves were built up, amplifying cDNAs synthesized from serial dilutions (from 1 to 1000 ng) of total RNA. Linear equations, determination of coefficient (R^2^), and reaction efficiencies were calculated. cDNAs were synthesized by reverse-transcribing 500 ng of total RNA using the iScript cDNA Synthesis Kit (Bio-Rad Laboratories, Hercules, CA, USA) and amplified in a 20 µL mixture volume containing 10 µL of Sso Advanced SYBR Green Supermix (BioRad), 0.5 µL (0.5 µM final concentration) of each primer, 8 µL of nuclease-free water, and 1 µL of cDNA. In negative control samples, cDNA was replaced by ultra-pure water or total RNA to detect possible contaminations. Reaction conditions were run in a CFX96 Touch Real-time PCR Detection System (Bio-Rad) using the following conditions: 95 °C for 5 min and 40 cycles of 94 °C for 20 s, 60 °C for 20 s, and 72 °C for 20 s. Data were acquired using CFX-Manager Software v1.6 (Bio-Rad Laboratories). Melting curves were obtained at temperatures ranging from 65 to 95 °C. Acquisition was performed at every 0.5 °C increase in temperature, with a 10 s step. Differential expression was determined according to the 2^−ΔΔCq^ method [27,28]. Actin was used as a reference gene (Table 2).

## 3. Results and Discussion

### 3.1. Analysis of RNA-Seq Data

RNA-sequencing generated a total of 88,806,346 raw reads, of which 99% (87,765,686) passed quality filtering with a sequence quality score ≥ 30. The high-quality reads were aligned to the *V. vinifera* reference genome, achieving an average mapping rate of 98.83%. Unique reads that aligned with the reference coding gene set were utilized for downstream analyses to evaluate the similarity among the eight samples using Euclidean distance based on rlog-transformed data. This analysis revealed clear clustering of samples according to treatment (CTR vs. treated) and sampling time (6 and 24 HPT), reflecting distinct expression profiles (Table S1 and Figure S1).

### 3.2. Comparative Analysis of Gene Expression at 6 and 24 HPT

**Figure 1:**
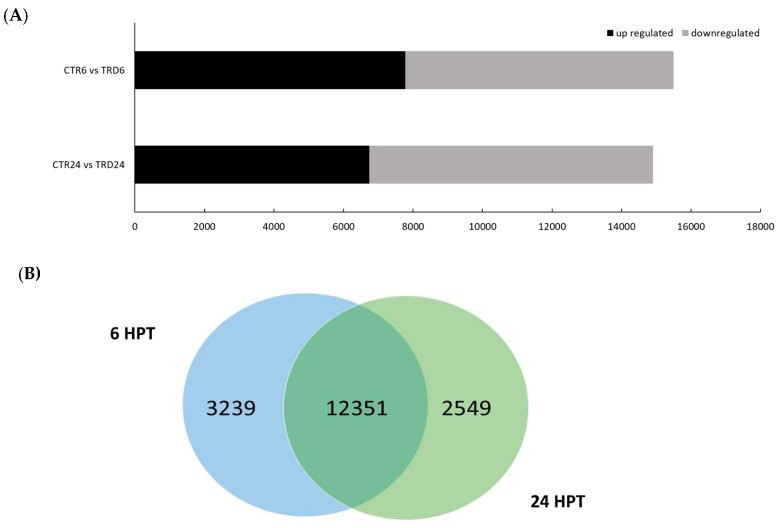
(**A**) Up-/downregulated genes (FC ≥ 2 and FDR < 0.05) at 6 vs. 24 HPT. (**B**) Venn diagram of genes modulated in pairwise time point comparisons between 6 and 24 HPT by soybean hydrolysate application in *Vitis vinifera* leaves.

The modulation of the differentially expressed genes (DEGs) following hydrolysate treatment of *V. vinifera* cv. Corvina was observed at two time points (6 and 24 HPT) to more closely study the differences in the modulated genes following the application of the biostimulant formulation. Notable findings emerged from pairwise comparisons between untreated (CTR) and treated samples (TRD) at both time points (CTR6 vs. TRD6 and CTR24 vs. TRD24), highlighting host responses under identical environmental conditions. A substantial number of DEGs was recorded between the first and second time points (Figure 1A). The number of DEGs was slightly higher at 6 HPT, with 15,495 genes, compared to 14,910 DEGs at 24 HPT. A higher number of upregulated genes compared to downregulated genes was observed at 6 HPT, while analysis at 24 HPT showed the opposite behavior. Among these, 3223 genes were uniquely regulated at 6 HPT, while 2638 were specifically modulated at 24 HPT (Figure 1B).

The identified DEGs were subsequently categorized according to MapMan functional groups, with the sixteen most abundant categories summarized in Table 3. With more than 500 modulated genes, the most abundant categories, “stress”, “signalling”, “secondary metabolism”, “transport,” and “plant and growth development”, were considered to elucidate the resistance induction effect and showed comparable representation at both time points.

Assumptions made in this overview section will be further discussed in the following paragraphs considering the “time-effect” in treated samples vs. untreated control, since—in our opinion—this not only provides a comprehensive overview of the response of *V. vinifera* plants cv. Corvina to biostimulant application but also highlights the specific traits enabling the early response which allows plants to better adapt to stress conditions. The discussion will include the analysis of transcripts involved in different relevant biological pathways, such as pathogen perception and stress responses, secondary metabolism, transport, and plant growth development.

### 3.3. Main Processes/Defense Pathways Underlying Differential Behavior in Response to Formulate Application

#### 3.3.1. Pathogen Perception and Stress Responses

A more detailed investigation within the category of response to biotic stress revealed an interesting and abundantly represented group of DEGs belonging to the PRs protein family presenting NBS-LRR domains, namely Resistance Gene Analogs (RGAs), disease resistance proteins (RPSs and RPP13), Dirigent-like protein (DIRs), and disease resistance response protein 206 (DRR206). For each member, isomeric forms were present. Plants use R genes to recognize pathogens and activate defense signaling pathways. Most R proteins contain nucleotide-binding sites and leucine-rich repeat (NBS-LRR) domains [31,32,32,33,34]. Studies suggested that RGAs were promising candidate resistance genes also against downy mildew, as they shared conserved motifs, resembled known R proteins, and were induced after infection [25]. In a recent study, several RPP-like, RPS2-like, and RGA4 proteins were annotated as disease resistance proteins in grapevine [35].

1. RGA2 appeared to be the most represented in our libraries. Once activated, RGA2 initiates signaling cascades that generate reactive oxygen species (ROS) and calcium influx, leading to a hypersensitive response and reinforcement of cell walls, ultimately enhancing grapevine immunity [36]. We observed that RGA genes were strongly upregulated at 6 HPT, whereas they were mostly downregulated at 24 HPT.
2. RPS genes have been classified within the functional category of signal transduction and environmental adaptation, highlighting their central role in perceiving external stress signals and activating downstream defense pathways [37]. In fact, a specific resistance locus, designated as RPS2, was associated with the recognition of *Pseudomonas syringae* strains expressing the a*vrRpt2* avirulence gene [38]. We observed that RPS genes were upregulated at 6 HPT, whereas they were downregulated at 24 HPT.
3. RPP13, a member of the cytoplasmic class of disease resistance genes, encodes one of the most variable *Arabidopsis* proteins identified so far [39]. It was observed that RPP13 in *A. thaliana* and tobacco enhanced resistance to oomycete pathogens, and this enhancement is closely related to the activation of the salicylic acid (SA) signaling pathway [39].
4. The DRR206 protein was also a representative element in our study. The DRR206 gene was strongly induced in pea in response to both fungal and bacterial pathogens and elicitors. It was reported that constitutive expression of DRR206 can confer substantial resistance to blackleg disease in pea [40,41].
5. DIR play important roles in the biosynthesis of lignin and lignans and are involved in various processes, such as plant growth, development, and stress responses. During pathogen infections, they contributed to enhancing plant resistance by promoting lignin accumulation [42]. However, there is little information about VvDIR proteins in grapevine. In our case, the gene was strongly upregulated at 6 HPT and slightly downregulated at 24 HPT.

Both RGA and RPS patterns suggest that they could play a more active and effective role in pathogen defense during the early stages of infection compared to later stages following biostimulant application. In our study, RPP13 was only upregulated at 6 HPT; this suggests that its function might be involved in early stages of defense response following biostimulant application. In our study, pDIR9 was differentially expressed at both time points, indicating that it might be involved in multiple plant pathways.

The results of our study provide valuable insights into the roles of key genes in the plant immune response following application of the biostimulant formulation. RGA and RPS genes were strongly upregulated during the early stages of AgriD application, suggesting their crucial role in initiating defense mechanisms. Similarly, RPP13 and DRR206 appeared to be most effective during the initial phase of defense, with their expression peaking at 6 HPT before gradually declining. These findings highlight the importance of early defense responses in pathogen resistance, where specific genes were activated to rapidly recognize and respond to pathogens. On the other hand, pDIR9 showed differential expression at both early and later time points, indicating its involvement in multiple plant defense pathways and suggesting that it may serve a broader regulatory role across the immune response.

Together, these data underscore the potential of biostimulants to activate early defense mechanisms in plants and highlight the importance of specific genes in coordinating these responses. Understanding the temporal dynamics of gene expression in response to formulate application can provide valuable insights for improving plant resistance strategies and developing more effective biostimulants for ameliorating crop responses to stresses.

Many proteins belonging to this category and present in our libraries were also involved in abiotic stress.

6. One of the most abundant protein classes was Germin-like proteins (GLPs). They are glycoproteins closely associated with plant development and biotic and abiotic stress responses in the plant kingdom. *V. vinifera* has seven GLP members, from VvGLP1 to VvGLP 7, of which VvGLP3 proved to be induced in response to powdery mildew in epidermal cells [43]. In another work, many GLP genes on the 4A, 4B, and 4D chromosomes of wheat showed increased expression during powdery mildew infection [44]. With regard to abiotic stresses, CsGLP1-6 and CsGLP7-1 were found to be upregulated under salt and drought stresses but downregulated following ABA treatment, indicating that these genes may play positive roles in mediating salt and drought stress responses through ABA-dependent signaling pathways [45]. In our transcriptomic analysis, these genes were upregulated at both 6 and 24 HPT, with higher expression observed at 24 HPT.
7. Thaumatin-like proteins (TLPs) form a large family in plants, with members performing diverse functions in response to biotic and abiotic stresses. In fact, it belongs to the PR5 subgroup that is particularly associated with defense against pathogens and environmental challenges such as cold, salt, and drought stresses [46]. In our analysis, TLPE22 was upregulated only at 6 HPT.
8. EF-Tu Receptor (EFR) is a plasma membrane resident receptor responsible for recognition of microbial elongation factor Tu (EF-Tu) and thus triggering plant innate immunity to fend off phytopathogens. Functional EFR must be subjected to endoplasmic reticulum quality control (ERQC) machinery for correct folding and proper assembly to reach its destination. More recent studies by two independent research groups have revealed that a set of ERQC components, including Early Responsive To Dehydration (ERD2b and ERdj3b), binding protein precursor (BIPs), and Taurosporin and Temperature-Sensitive 3-Like A (STT3a) proteins were required for the generation of the functional EFR [47]. In our transcriptomic analysis, STT3a, BIP, and ERD genes were differentially expressed at both time points, indicating their potential involvement in the coordination and quality control processes of the ERQC system. ERD4 was also found in the transcriptome analysis of *V. amurensis* under cold stress, where it only increased during cold treatment [48]. ERDs genes were regulated at both time points.
9. Heat stress transcription factors (HSFs) are central regulators of the heat stress response, where they are recognized as main chaperone components against unfolded protein accumulation [49]. In recent years, HSFs proved to be involved in the control of a multitude of stress responses and in developmental processes. HSFB2a was related to heat response and seedling size and was required for plant fertility [50]. Also, a recent study discussed the role of the module E3 ligase XB3 ortholog 1 XBAT31-HSFB2a/B2b in conferring reproductive thermotolerance in plants [51]. The HSP family was significantly represented in our libraries, and it was upregulated at 6 HPT but downregulated at 24 HPT.

To summarize, GLPs may exhibit prolonged activity and effectiveness during later stages following biostimulant application. In contrast, TLPs may exhibit activity and effectiveness during the early stages following biostimulant application, as well as HSPs. Finally, ERDs may exhibit prolonged activity and effectiveness from the early to the later stages following biostimulant application. In summary, these findings indicate that AgriD application triggered a covered and time-dependent activation of abiotic stress-related genes. Early responders, like TLPs and HSPs, set up the plant for immediate stress, while GLPs and ERDs maintained and extended protective effects into later stages, contributing to a more robust and sustained stress tolerance profile. This matched gene expression pattern highlighted how biostimulants can adjust plant stress responses to enhance resilience across different phases of stress exposure.

**Figure 2:**
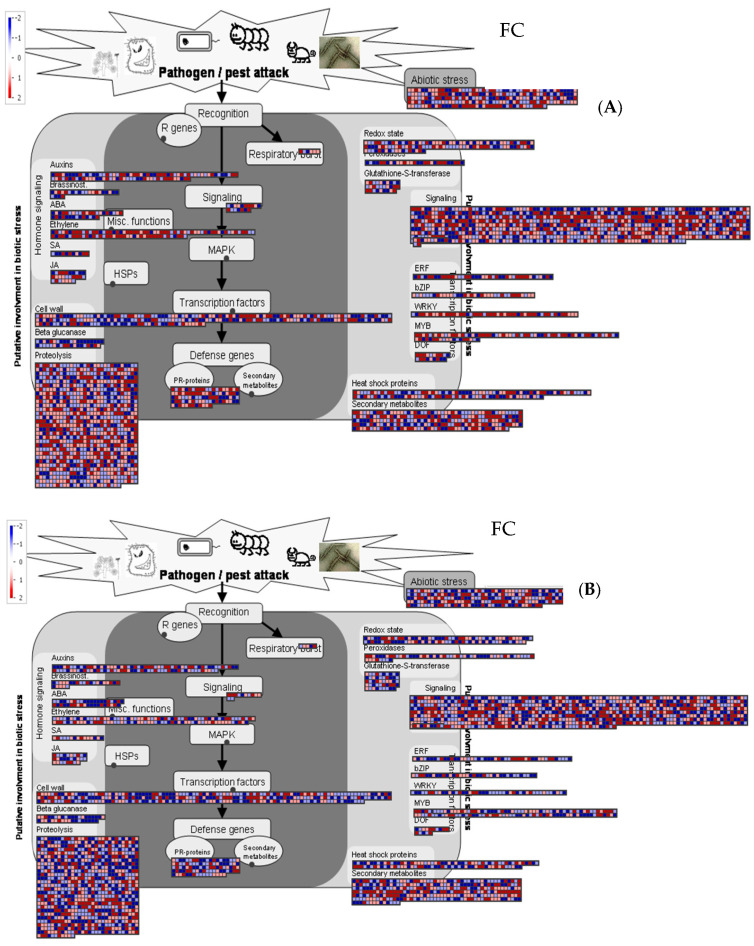
The response of *Vitis vinifera* to AgriD biostimulant application involved the modulation of pathways associated with plant environment interactions at 6 (**A**) and 24 HPT (**B**) and visualized using MapMan software. Absolute values of fold change (ratio treatment/control) >2 was established as a threshold to judge the significance of gene expression difference. Blue indicated a decrease, whereas red indicates an increase (see color scale).

All transcriptomic data can be effectively represented using MapMan pathway visualization, which enables the integration and intuitive interpretation of large sets of differentially expressed genes. As shown in Figure 2A,B, the analysis highlighted responses related to plant–environment interactions at 6 and 24 HPT (Table S2). The MapMan representation not only revealed which functional categories were modulated but also illustrated the magnitude and direction of these transcriptional shifts, providing a comprehensive and coherent overview of the major biological processes activated under the examined conditions.

#### 3.3.2. Secondary Metabolism

Transcriptomic analyses revealed the presence of genes associated with phenylpropanoid, flavonoid, stilbene, terpenoid, and phytoalexin biosynthesis pathways. These compounds are key secondary metabolites related to plant defense and stress responses, indicating that such pathways may be up- and downregulated under the studied conditions [52]. In a comparative transcriptomic analysis between unripe and ripe *V. flexuosa* berries, several genes showed differential expression patterns that were also reflected in our transcriptomic data, i.e., laccases (LAC), phenylalanine ammonia-lyase (PAL), cinnamyl alcohol dehydrogenase (CAD), chalcone synthase (CHS), isoflavone reductase (IFR), and terpene synthase (TPS) [53].

Isoprenoids or terpenoids are a large and diverse class of secondary metabolites that play key roles in plant defense, signaling, and adaptation [54]. Monoterpenes and sesquiterpenes are major volatile compounds released after herbivore damage, attracting natural enemies of herbivores and reducing further damage. Beyond volatiles, certain diterpenes and sesquiterpenes act as phytoalexins, providing direct defense against herbivores and microbial pathogens [54,55]. The proteins listed above in cooperation with mevalonate kinase (MVK), S-alkyl-L-cysteine (allicin), and cycloartenol synthase 1 (CAS1) were representative in our transcriptomic analysis.

In fact,

1. TPSs, like TPS14 and TPS21, are members of the gene family, which catalyzes the formation of terpenoids (e.g., linalool, geraniol, and nerol). The sequencing and assembly of a grapevine inbred Pinot noir genome (PN40024) led to the prediction of 89 grapevine TPS (VvTPS) genes involved in terpenoid secondary metabolism [56]. Furthermore, in *Solanum lycopersicum,* several transcripts like TPS14 were found expressed specifically in flowers, whereas TPS21 was detected in flowers, roots, green fruits, leaves, and stems [57]. According to our data, the TPS21 gene was upregulated at 6 HPT but downregulated at 24 HPT, whereas TPS14 exhibited the opposite behavior.
2. LAC17 and LAC14 are multicopper oxidases involved in oxidizing phenolic compounds, lignin polymerization, secondary cell wall formation, and plant defense. LAC17 was reported to contribute to lignin polymerization in *A. thaliana* [58]. These genes were downregulated in ripe berries compared to unripe berries of *V. flexuosa*, ultimately resulting in a better response to stresses [53]. In our transcriptomic analysis, LAC14 and LAC17 were more strongly modulated at 6 HPT as compared to 24 HPT; in particular, LAC14 was more highly upregulated compared to LAC17.
3. PAL is the first enzyme in the phenylpropanoid pathway, including phytoalexin biosynthesis, converting L-phenylalanine in trans-cinnamic acid, which is involved in plant growth, development, and response to stresses [59]. It was reported that quadruple mutants of the PAL gene family accumulated substantially reduced levels of salicylic acid in *Arabidopsis* and displayed increased susceptibility to a virulent strain of the bacterial pathogen *Pseudomonas syringae* [59]. In our transcriptomic analysis, the PAL3 gene was highly upregulated at 6 HPT, but its expression was slightly downregulated at 24 HPT. This finding is in agreement with our previous investigation that reported a significantly higher resveratrol accumulation at 4–8 HPT, followed by a marked decrease at 24–72 HPT [15].
4. IFR is a key enzyme in the phenylpropanoid pathway and plays a critical role in plant defense, especially in legumes and other plants that produce isoflavonoid phytoalexins [60]. PvIFR (EC 1.3.1.45) is reported to play a complex role during plant growth and root development, involving auxin transport [61]. In chickpea, IFR levels increased in response to fungal infection, indicating a possible role in disease resistance [62]. The observed increase in IFR levels during fungal infection supports its involvement in plant immunity, making it a potential target for improving disease resistance in crops. In our transcriptomic analysis, IFR genes were strongly upregulated at 6 HPT, while they were downregulated at 24 HPT.
5. CHS (EC 2.3.1.74) is a key enzyme of the flavonoid/isoflavonoid biosynthesis pathway and is a member of the plant polyketide synthase superfamily, which also includes STS. CHS plays a vital role in flavonoid synthesis, including antimicrobial molecules like phytoalexins [63], influencing plant growth, development, and response to both biotic and abiotic stresses [64]. It was observed that CsCHS genes effectively restored flavonoid production in *Arabidopsis chs*-deficient mutants. Additionally, CsCHS-transgenic tobacco plants exhibited higher flavonoid accumulation compared to their wild-type counterparts [65]. Stilbenes are central phytoalexins in *V. vinifera*, playing a pivotal role in disease resistance [66]. The STS allele from the wild Chinese grapevine, expressed in *Arabidopsis* as a heterologous system, was proven to cause accumulation of stilbenes and increase resistance against the powdery mildew agent *Golovinomyces cichoracearum* [67]. Thus, the induction of stilbene biosynthesis might contribute to the basal immunity against the downy mildew of grapevine, thus representing an adaptive resistance trait to recover, in both cultivated and wild *V. vinifera* germplasm [20]. In our transcriptomic analysis, CHS and STS family proteins were upregulated at 6 HPT, while their expression decreased at 24 HPT [15].
6. Mevalonate kinase (MVK or MK) plays a central role in the mevalonate (MVA) pathway, which is one of the two major routes (alongside the MEP/DOXP pathway) for isoprenoid biosynthesis in plants [68], including grapevine [69]. A study showed that MVK enzymes were upregulated during elicitor-induced sesquiterpene phytoalexin accumulation in rice [70]. Also, MVK interacts directly with the plant extracellular ATP (eATP) receptor P2K1 and is phosphorylated by P2K1 in response to eATP. Mutation of P2K1-mediated phosphorylation sites in MVK eliminates the ATP-induced cytoplasmic calcium response and MVK enzymatic activity and suppresses pathogen defense [71]. In our transcriptomic analysis, this enzyme was more strongly upregulated at 24 HPT as compared to 6 HPT, when its expression was lower.
7. Nitric oxide (NO) is an important signaling molecule involved in regulating plant responses to abiotic stress [72]. In garlic, S-alkyl-L-cysteine (SAC; EC 4.4.1.4) is a pyridoxal-5′-phosphate-dependent enzyme that rapidly converts the stable precursor alliin into the biologically active compound allicin upon tissue disruption. Allicin and related thiosulfates display potent antimicrobial activity by disrupting microbial membranes, inhibiting thiol-containing enzymes, and inducing oxidative stress [73]. Although SAC is known to induce NO synthesis in animals, its behavior in plants remains poorly understood. However, recent evidence has shown that spraying rice seedlings with SAC increases NO synthesis, modulates the expression of genes involved in Cd transport, enhances Cd sequestration in root cell wall components, and reduces Cd accumulation in both roots and shoots [72]. SAC also contributes to the formation of important sulfur-containing volatiles from cysteine–S conjugate precursors. This enzymatic family has been isolated from animals to microorganisms and plants like grapevine. Quantification of such precursors in grapes or musts could allow for the assessment of the aromatic potential of several grapevine cultivars as quality control [74]. In our study, this enzyme was more highly represented and upregulated at 24 HPT compared to 6 HPT, where its differential expression was lower.
8. Plant sterols are vital components of cell membranes and lipid rafts, essential for plant development and stress tolerance. Cycloartenol synthase 1 (CAS1) is a key enzyme in the sterol biosynthetic pathway, responsible for producing membrane sterols and precursors of bioactive metabolites. Studies have shown that CAS1 significantly influences sterol biosynthesis, as well as other physiological and biochemical processes, including pigment formation and overall plant growth regulation [52]. In our transcriptomic analysis, CAS1 was upregulated at 6 HPT, while it was slightly downregulated at 24 HPT.

These results regarding TPS genes suggest that plants respond dynamically to treatment with the formulate, actively modulating terpene biosynthesis and potentially enhancing terpenoid production compared to untreated controls. These findings suggest that LAC genes might be more effective during the very early stages of the response to treatment application, especially in the induction of the structural barriers that might contrast fungal penetration and colonization than at a later stage. These results suggest that PAL3, IFRs, CAS1, CHS, and STS genes played an important role during the early stages of the biostimulant application, while SAC and MVK followed the opposite trend. Activation of secondary metabolism is a key element of the plant response to biostimulant application. In our study, the expression patterns of several secondary metabolism-related genes highlighted a very dynamic and stage-specific response to the treatment. TPS genes, involved in terpene biosynthesis, were strongly activated, suggesting that AgriD stimulated the production of terpenoids, compounds widely known for their antimicrobial, defensive, and signaling functions. LAC genes, associated with lignin formation and cell wall strengthening, were particularly responsive in the very early stages, indicating a rapid reinforcement of structural barriers that could limit fungal penetration and colonization. Similarly, PAL3, IFRs, CAS1, CHS, and STS genes, all central to phenylpropanoid and stilbene biosynthesis, were strongly induced early on, reinforcing the idea that plants quickly invested in protective phenolic and phytoalexin pathways. On the contrary, SAC and MVK showed the opposite trend, suggesting that not all secondary metabolic branches respond similarly and that some pathways may become more relevant at later stages or under different physiological needs. This coordinated activation likely enhanced plant alertness and resilience, contributing to improved defense and performance under stress.

**Figure 3:**
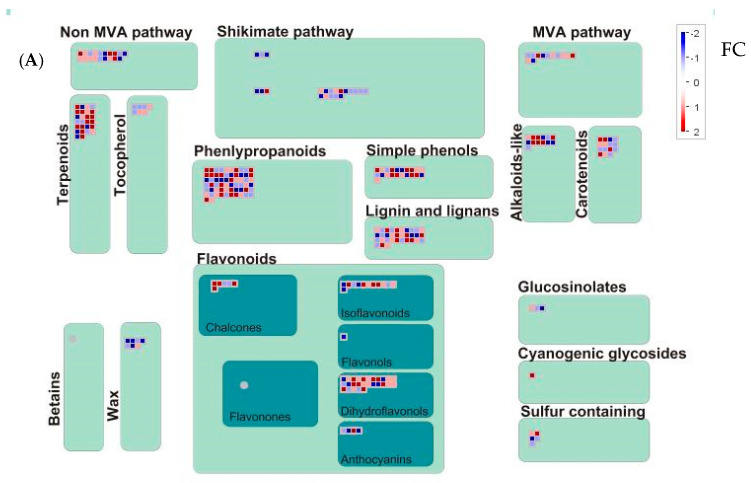

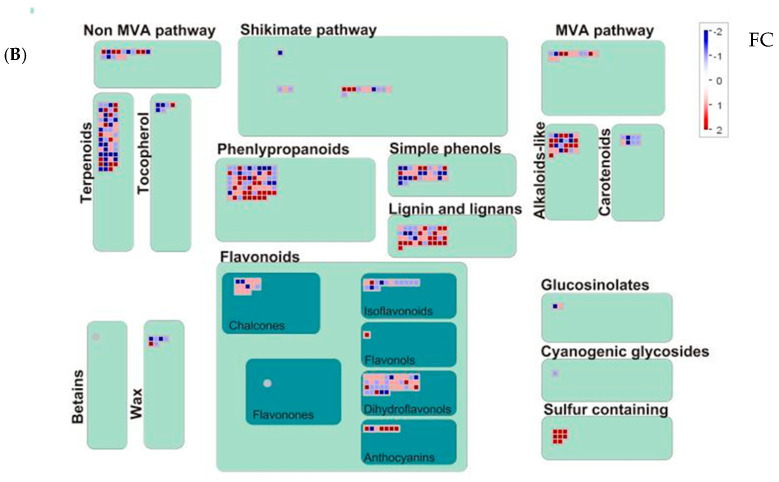
The response of *Vitis vinifera* to AgriD biostimulant application involved the modulation of pathways associated with secondary metabolism at 6 (**A**) and 24 HPT (**B**), visualized using MapMan software. Absolute values of fold change (ratio treatment/control) >2 was established as threshold to judge the significance of gene expression difference. Blue indicates a decrease, whereas red indicates an increase (see color scale).

As described in the “*Pathogen Perception and Stress Responses*” chapter, interactions among secondary metabolism pathways can likewise be visualized using MapMan software. In this context, Figure 3A and Figure 3B illustrate the transcriptional changes observed at 6 and 24 HPT, respectively (Table S3).

#### 3.3.3. Transport, Plant Growth, and Development

Plant growth and development depend on well-coordinated metabolic, signaling, and transport processes. The movement of water, nutrients, and metabolites across cells and tissues is essential for maintaining homeostasis and enabling adaptation to environmental changes. Membrane transporters are central to these processes, connecting metabolism with energy production, biosynthesis, and stress responses [75]. In our transcriptomic analysis, the most representative gene family identified was Nodulin family transporters (MtN21). Besides MTN21, other proteins actively involved in the transport process were present, such as Sodium Hydrogen Exchanger (NHXs), Cation/H^+^ Exchanger Transporters (CAXs), and Natural Resistance-Associated Macrophage Protein 3 (NRAMP3s). Meanwhile, proteins involved indirectly in transport included Sulphur Deficiency-Induced 1 (SDI1s), Caleosins (CLOs), Responsive to Desiccation 26 (RD26), and Senescence-Associated Gene101 (SAG101) (Table S4).

Actually,

1. MtN21 genes encode plant-specific transporter proteins involved in diverse physiological and developmental processes, particularly those related to symbiosis, nutrient transport, and stress adaptation. In a recent work, mutants of *MtUMAMIT14* (from the MtN21 family) in *Medicago truncatula* showed impaired nodule formation and nitrogen fixation [76]. A recent review emphasized the emerging role of nodulin-like proteins as transporters of nutrients, solutes, amino acids, and hormones in non-modulating plant species, including *V. vinifera* [77]. In our transcriptomic analysis, MtN21 was upregulated at 6 HPT, while at 24 HPT, its expression was slightly downregulated.
2. NHXs are intracellular Na^+^/H^+^ antiporters that play key roles in maintaining cellular pH and Na^+^ and K^+^ homeostasis. NHX1 and NHX2 proved to be crucial for cell growth and flower development by regulating vascular potassium levels and pH, which in turn control cell expansion in *A. thaliana* [78]. Plants with a double-knockout *nhx1 nhx2* exhibited abnormal flowers, failed to produce siliques, had shortened hypocotyls, and displayed heightened sensitivity to external potassium [78]. Additionally, NHX1 expression was strongly induced under high salinity, promoting the sequestration of Na^+^ into vacuoles, which lowers cellular osmotic potential and helps protect the cell from ion toxicity [79]. Even if genomic and functional studies in distinct species were conducted, the grapevine NHX family has not been described yet. However, through genome-wide identification, molecular characterization, and gene expression analysis in grapevine, all VvNHXs were reported to participate in berry growth, except VvNHX5, which was implicated in seed maturation, while VvNHX4 could be more involved in floral development. VvNHX1 played a crucial role in several grape developmental steps and adaptation responses through mechanisms of phyto-hormonal signaling [80]. In our transcriptomic analysis, NHX2 genes were markedly induced at 24 HPT (up 123-fold), while their expression was minimal at 6 HPT (up 2-fold).
3. CAXs are another important class of ion transporters, namely “proton–cation exchangers”, which belong to one of the five related transporter families that, together, form a large phylogenetic clade [81,82]. In grapevine, *Vv*CAX3 mediates calcium transport across the vascular membrane and was detected in stems, leaves, roots, and berries, with a peak at the pea-size stage, followed by a gradual decline throughout the maturation process. The VvCAX3 promoter contains several predicted cis-acting elements associated with developmental regulation and stress response processes [83,84,85]. Like NHX, CHX was strongly induced at 24 HPT, whereas its expression remained minimal at 6 HPT.
4. NRAMP3 is a metal transporter belonging to the NRAMP family, which mediates the movement of divalent metal ions across cellular membranes. In plants, NRAMP3 played essential roles in metal homeostasis, seedling development, and stress responses [86]. Furthermore, the expression of NRAMP1 and NRAMP3 in response to copper suggested that these transporters might play an important role in heavy metal tolerance in grapevine rootstocks [87]. Like previous protein transporters, the NRAMP3 gene was strongly induced at 24 HPT, while its expression was low at 6 HPT.
5. The SDI1 gene plays an important role in plant metabolism and development under conditions of limited sulfur availability [88]. The expression of SDI1/SDI2 genes was reported to play a negative role in the biosynthesis of non-essential sulfur-containing compounds (such as glucosinolates) but a positive role in reallocating resources and promoting the plant’s metabolic adaptation under sulfur deficiency conditions [89]. SDI1 was upregulated at 6 HPT, while it was downregulated at 24 HPT.
6. CLOs are lipid-associated proteins that play various physiological roles in plant growth, development, and plant–environment interactions like abiotic and biotic stresses [4]. It was possible to identify 922 types of caleosins from 203 plant species. In a recent work, it was demonstrated that Caleosin was implicated in development and salt tolerance in rice [90]. Also, CLO3, a gene strongly induced by drought, salt, and abscisic acid, interacted with G-protein signaling and was shown to affect growth and morphology in *A. thaliana* [91]. In our transcriptomic analysis, CLO-related family protein was upregulated at 6 HPT, while its expression decreased at 24 HPT.
7. RD26 is a NAC-domain transcription factor known in *A. thaliana* for regulating stress responses, leaf senescence, and hormone signaling [92]. In grapevine, RD26-like genes also played roles in development and abiotic stress tolerance, especially drought. In fact, overexpression of *Va*NAC26 (from *V. amurensis*) in *A. thaliana* improved drought tolerance and affected ROS and JA synthesis [93]. In addition, *Vv*NAC26 has been related to the early development of grapevine inflorescences and berries [94]. In our transcriptomic analysis, ND26 was strongly induced at 6 HPT, while its expression decreased at 24 HPT.
8. SAG101 was first identified for its role in cell membrane degradation during leaf senescence in *Arabidopsis* [95] and later reported to play roles in plant immunity and abiotic stress response via interaction with EDS1 and PAD4 [96]. However, PagSAG101 in *Populus* L., an ortholog of *Arabidopsis*, played an important role in plant development, particularly wood formation [97]. In our analysis, this gene was strongly upregulated at 6 HPT, while its expression decreased at 24 HPT.

Genes encoding membrane transporters showed a minimal response at 6 HPT but were strongly activated at 24 HPT, indicating that transporter functions belong to a secondary response phase supporting later physiological adjustments triggered by the biostimulant. In contrast, genes involved in plant development were rapidly upregulated at 6 HPT but downregulated by 24 HPT, suggesting an early, short-lived activation of growth-related pathways followed by a shift toward other priorities such as stress adaptation or metabolic reprogramming. Overall, these contrasting temporal patterns highlight a coordinated transition from early developmental responses to later transporter-mediated processes in the plant’s reaction to the biostimulant application.

The data discussed refer to the biostimulant application in standardized greenhouse conditions on the grapevine cv. Corvina as a model cultivar to highlight the pathway specifically triggered by the treatment. However, a future study should focus on comparing the response of different grapevine cultivars and terroirs to the application of the same biostimulants, as it may vary depending on the conditions to which the plant is exposed [10].

### 3.4. Expression Analysis of the Selected Candidate Genes

To validate the libraries obtained by RNASeq, RT-qPCR was performed on five genes randomly selected for high or low expression levels. Linear equations, determination coefficients (R^2^), and reaction efficiencies for all the primer pairs are reported in Table 4. Reaction efficiencies were similar and included in the optimal range of 90–110%. The determination coefficient was comparable and close to 1. Relevant genes selected for the present study were phenylalanine ammonia lyase (PAL), stilbene synthase (STS), chitinase 3 (CHIT3), β-1,3-glucanase (GLUC), polygalacturonase inhibiting protein (PGIP), and the housekeeping actin (ACT).

**Figure 4:**
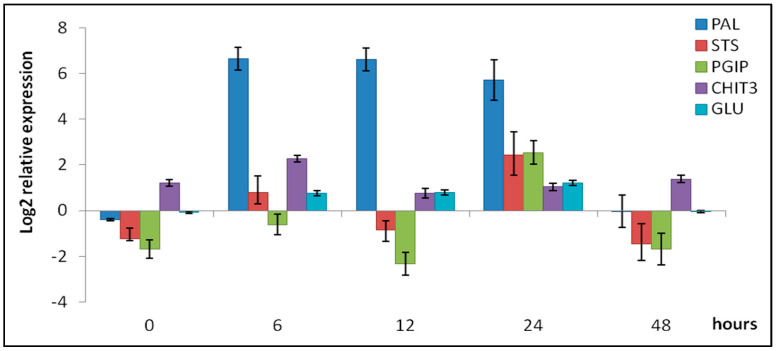
Relative gene expression of phenylalanine ammonia lyase (PAL), stilbene synthase (STS), polygalacturonase inhibiting protein (PGIP), chitinase 3 (CHIT3), and β-1,3-glucanase (GLUC) genes in grapevine leaves in response to AgriD biostimulant from 0 to 48 HPT. Data were analyzed using the 2^−ΔΔCt^ method and normalized using the actin gene. The data represent the mean of 3 replicates ± standard error of the mean (SEM).

The expression patterns observed in the RNA-Seq dataset were consistently validated by the qPCR results (Figure 4). The PAL gene exhibited the highest level of upregulation between 6 and 24 HPT, whereas its expression became undetectable at 48 HPI. Although the STS gene showed a variable expression trend, it was upregulated at 6 and 24 HPT. A comparable pattern of co-expression of the two genes has been documented in *V. vinifera* cv. ‘Optima’ cells treated with fungal cell wall components [85] and oligosaccharides [98] upon treatment with pathogen-derived elicitors. The transcriptional changes included the induction of a protein synthesis inhibitor, PGIP, which is capable of inhibiting fungal endo-polygalacturonases (PGs) [99]; PGIP was upregulated only at 24 HPT by a 6-fold increase compared to the control. Its role in plant defense has been demonstrated; in fact, the upregulation of PGIPs in transgenic *Arabidopsis* plants proved to confer enhanced resistance to *B. cinerea* [29,100]. Furthermore, previous studies have reported the induction of PGIP in grapevine tissues in response to elicitor treatments, including laminarin and oligogalacturonides [30,101]. The qPCR analysis confirmed the induction of genes encoding various PR protein families with antimicrobial activity, including chitinases and β-1,3-glucanases, which play key roles in grapevine defense responses [29,102]. Indeed, the relative expression values of CHIT3 and GLU were 2.05- and 2.31-fold, respectively.

## 4. Conclusions

Grapevine is a globally significant crop that is facing growing challenges under intensifying biotic and abiotic stresses. As traditional management strategies have become increasingly unsustainable, the need for environmentally friendly alternatives has become evident. Among these, biostimulants have emerged as promising tools to enhance plant performance and resilience. The obtained data demonstrate that the biostimulant formulation application can activate key defense responses and other metabolic pathways in grapevine (cv. Corvina), potentially equipping plants with an improved capacity to withstand potential stressing conditions. Indeed, several important protein families were abundantly identified, belonging to categories related to transport, plant growth and development, stress response, and secondary metabolism. Several protein categories were found to be activated during early hours following biostimulant application (6 HPT), underscoring their relevance in the initial phases of the plant’s response to stress. Conversely, other proteins were activated after a longer period (24 HPT), reasonably being involved in a secondary response. Additionally, certain proteins exhibited sustained activity over an extended timeframe (from 6 to 24 HPT). By integrating all the results, we may hypothesize a faster and more effective reaction by the plant to stress conditions 6 h after biostimulant exposure compared with 24 h. These findings support the application of biostimulants not as a one-off “silver bullet,” but rather as a component of a sustainable management strategy that requires timely and repeated applications [103], possibly combined with other agronomic practices, to maintain plant vigor, resilience, and yield under changing environmental conditions. Among our future objectives is the extension of this approach to other crops of global and agronomic significance, as well as the evaluation of novel plant-derived biostimulants for an integrated preventive strategy. The main goal will be to contribute to minimizing the use of synthetic pesticides and fertilizers while promoting sustainable crop management strategies.

## Figures and Tables

**Table 1:** Summary of cDNA libraries of *Vitis vinifera* treated or not treated with biostimulant.

Treatments	Sampling Time (HPT)	cDNA Libraries	Total N. Reads *	Total Mapped Reads (%)	N. Mapped Reads Unique Match	Multi-Position Matches
Water (CTR)	6	CTR6	24,141,972	23,924,573 (99.1)	23,321,885	602,688
	24	CTR24	25,263,690	25,036,024 (99.1)	24,435,414	600,610
AgriD (TRD)	6	TRD6	22,999,933	22,565,185 (98.1)	21,773,766	791,419
	24	TRD24	16,400,751	16,239,904 (99.0)	15,893,962	345,942

* Total number of 50 bp reads that passed the quality check (QS ≥ 30).

**Table 2:** Selected genes and their primer sequences used for qPCR library validation.

Primers	Sequences (5′-3′)	Target Gene	Sources
PAL F	TGACCACTTGACTCACAAAT	Phenylalanine ammonia lyase	Bezier et al., 2002 [29]
PAL R	ACTAGGTATGTGGTAGACAT		
STS F	TACGCCAAGAGATTATCACT	Stilbene synthase	Bezier et al., 2002 [29]
STS R	CTAAAGAGTCCAAAGCATCT		
PR-3.4c F	GCAACCGATGTTGACATATCA	Chitinase 4	Aziz et al., 2003 [30]
PR-3.4c R	CTCACTTGCTAGGGCGACG		
PR3 F	AGATGGCATAGACTTCGACA	Chitinase 3	Aziz et al., 2003 [30]
PR3 R	GTACTTTGACCACAGCATCA		
PGIP-1 F	CCTAGACAATCCCTACATTC	Polygalacturonase	Bezier et al., 2002 [29]
PGIP-1 R	GACATTGGGGTCGAATCCTC		
PR-2.2	ATGCTGGGTGTCCCAAACTCG	Glucanase 2	Aziz et al., 2003 [30]
PR-2.2	CAGCCACTCTCCGACAGCAC		
VACT F	ATGTGCCTGCCATGTATGTTGCC	Actin	Aziz et al., 2003 [30]
VACT R	AGCTGCTCTTTGCAGTTTCCAGC		

**Table 3:** MapMan BINs (functional categories) of genes modulated in the untreated vs. treated samples at 6 and 24 HPT and number of DEGs assigned to each BIN.

BIN	BIN Name	No. of Corrected Clones per BIN (6 HPT)	No. of Corrected Clones per BIN (24 HPT)
1	PS	141	108
2	major CHO metabolism	84	77
3	minor CHO metabolism	86	92
4	glycolysis	42	38
5	fermentation	13	15
6	gluconeogenesis/glyoxylate cycle	8	9
7	OPP	19	14
8	TCA/org transformation	60	56
9	mitochondrial electron transport/ ATP synthesis	77	66
10	cell wall	258	304
11	lipid metabolism	263	246
12	N-metabolism	26	23
13	amino acid metabolism	194	171
14	S-assimilation	8	7
15	metal handling	39	43
16	secondary metabolism	246	314
17	hormone metabolism	324	367
18	co-factor and vitamin metabolism	52	50
19	tetrapyrrole synthesis	43	34
20	stress	552	541
21	redox	118	98
22	polyamine metabolism	13	12
23	nucleotide metabolism	120	105
24	biodegradation of xenobiotics	18	16
25	C1-metabolism	23	17
26	misc	850	1003
27	RNA	1842	1804
28	DNA	316	278
29	protein	2293	1998
30	signaling	867	843
31	cell	546	515
32	micro RNA, natural antisense, etc.	1	1
33	development	340	351
34	transport	700	672
35	not assigned	5091	4786
Σ		Σ 15,673	Σ 15,074
		mapped: 15,495	mapped: 14,910

**Table 4:** Linear equations, determination coefficients (R^2^), and reaction efficiencies obtained by plotting cDNA concentrations and Cq values achieved by qPCR of candidate genes.

Gene	Linear Equation	R^2^	PCR Efficiency (%)
ACT	Y = −3.583X + 30.671	0.99	92%
PAL	Y = −3.16X + 33.48	0.95	107%
STS	Y = −3.398X + 37.520	0.97	97%
PGIP	Y = −3.232X + 39.296	0.97	104%
CHIT3	Y = −3.583X + 41.991	0.98	90%
GLUC	Y = −3.445X + 35.520	0.98	95%

## Data Availability

The original contributions presented in this study are included in the article/Supplementary Material. RNA-Seq raw data were deposited in figshare (https://figshare.com/s/8451b9e2b78bd2bcece2, accessed on 14 January 2026). Further inquiries can be directed to the corresponding author.

## Supplementary Materials

The following supporting information can be downloaded at https://www.mdpi.com/article/10.3390/plants15020283/s1, Table S1: Functional annotation and differential expression; Table S2: “Pathogen perception and stress responses” category; Table S3: “Secondary metabolism” category; Table S4: “Transport, Plant growth and development” category. Figure S1. Two-dimensional principal component plot of RNA-Seq data from *Vitis vinifera* treated (TRD) or untreated (CTR) by biostimulant at 6 and 24 HPT.

## Abbreviations

The following abbreviations are used in this manuscript:

AgriD	AgricostanD
CTR	Control/water
ACT	Actin
BIP	Binding protein precursor
CAD	Cinnamyl alcohol dehydrogenase
CAS1	Cycloartenol synthase 1
CAX	Cation/H^+^ exchanger transporters
CHIT	Chitinase
CHS	Chalcone synthase
CLO	Caleosin
DIR	Dirigent-like protein
DRR206	Disease resistance response protein 206
EFR	EF-Tu receptor
EF-Tu	Microbial elongation factor Tu
ERD	Early responsive to dehydration
ERQC	Endoplasmic reticulum quality control
GLP	Germin-like proteins
GLUC	Glucanase
HPT	Hour post-treatment
HSP	Heat stress transcription factors
IFR	Isoflavone reductase
LAC	Laccases
MTN21	Nodulin family transporter
NHX	Sodium hydrogen exchanger
NO	Nitric oxide
NRAMP3	Natural resistance-associated macrophage protein 3
PAL	Phenylalanine ammonia-lyase
PGIP	Polygalacturonase
RD26	Responsive to desiccation 26
RGA	Resistance gene analogs
RPP13	Disease resistance proteins
RPS	Disease resistance proteins
SAC	S-alkyl-L-cysteine
SAG101	Senescence-associated gene101
STT3a	Taurosporin and temperature-sensitive 3-like A
TLP	Thaumatin-like proteins
TPS	Terpene synthase
TRD	Treated/AgriD
XBAT31	E3 ligase XB3 ortholog 1
