# Supplementary material for: Transcriptional Modulation in Grapevine by a Biostimulant Treatment for Improved Plant Resilience to Stress Events
Source: Plants (Basel). 2026 Jan 17;15(2):283. doi: 10.3390/plants15020283 (PMC12845150; doi:10.3390/plants15020283)
Supplement: Supplementary file 1 [file plants-15-00283-s001.zip › Figure S1.pptx]

## Slide 1
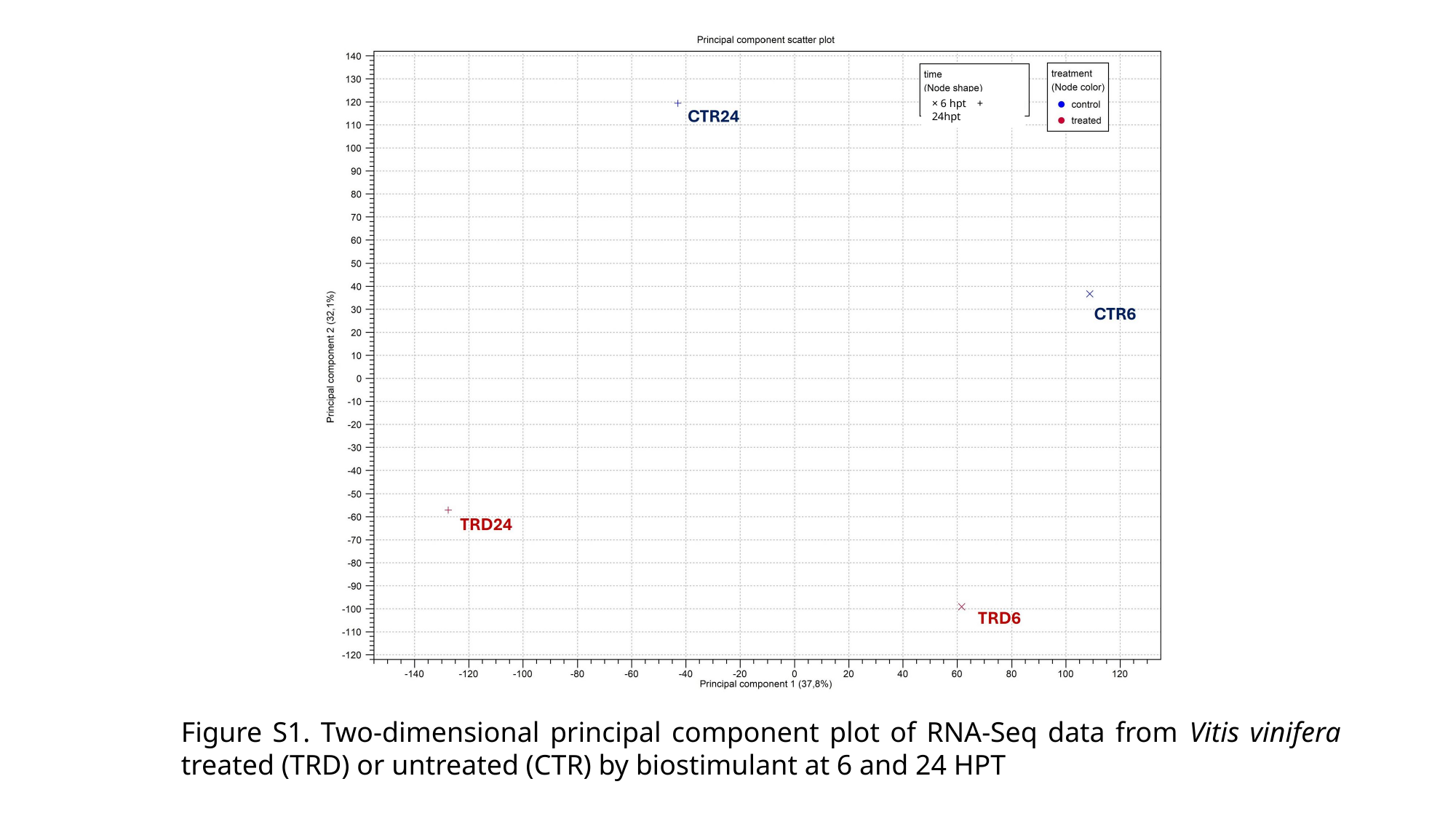

× 6 hpt + 24hpt
Figure S1. Two-dimensional principal component plot of RNA-Seq data from Vitis vinifera treated (TRD) or untreated (CTR) by biostimulant at 6 and 24 HPT
